# Medicine

**Published:** 1911-12

**Authors:** J. R. Charles


### Progress of the Medical Sciences.

### MEDICINE.

ATTENTION is drawn by Hort[1] to the very extraordinary diversity of opinion which exists at the present time as to the many practical points in the administration of tuberculin. Some competent observers hold that safe and efficient dosage should not exceed a small fraction of a milligramme of tuberculin T.R. Others report that they have given with safety and good results the stronger preparation T.O.A. in doses half a million times as great. Some maintain that tuberculin should only be used with the most careful clinical control; others that it may be used in relatively large doses with casual dispensary inspection.

Four kinds of reaction to tuberculin are known: (1) the local; (2) the focal, at the seat of the disease; (3) the general; (4) the reaction in vitro of certain of the body fluids, serum, blood, urine, milk, to certain artificially prepared reagents of the laboratory.

The local and focal reactions are the expression of an increased susceptibility of the tissues to the action of a substance to which before tubercular infection there was practically no susceptibility. These two reactions are a good example of the lowered resistance to the disease, on which increased resistance ultimately depends. Anaphylaxis is one of the body's greatest weapons in prophylaxis and cure. The detection of this anaphylactic state is the basis of the diagnostic use of the local and focal reactions produced by tuberculin administration.

It is the utilisation of this state which makes the therapeutic use of the tuberculin rational. On what exactly these local and focal reactions depend is at present unknown. With reference to the general action, it appears probable that this is due to the absorption of toxic material from the host's own cells which has been liberated owing to the action of the inoculated tuberculin on the focus of disease. A focal reaction is essentially a hyperæmia, and without this focal hyperæmia it is held that no general reaction can occur. Therefore an adequate dose of tuberculin in a tuberculous subject gives rise to a true auto-inoculation. The real goal of hetero-inoculation is successful auto-inoculation.

Tuberculin should not be used for diagnosis subcutaneously unless all other means of arriving at a diagnosis have been used, because (1) it is not always harmless even in the absence of such diseases as nephritis, cardiac disease or diabetes, all of which are held to be contra-indications to its use ; (2) a positive reaction may be obtained in quiescent tuberculosis ; (3) in advanced cases it often happens that no free so-called antibody necessary to the production of the reaction is present ; (4) there must be an absence of marked remittent or intermittent fever ; (5) there must also be an absence of syphilis, leprosy and actino-mycosis, since a reaction may occur in these diseases even when they are not complicated by tuberculosis ; (6) skilled clinical observation must be available for the detection of any slight increase in the physical signs in the focus of disease, as this evidence is one of the most valuable signs that a true reaction has taken place.

In using tuberculin treatment the aim should be directed towards the production of mild degrees of focal hyperæmia, which should be far below the plane of clinical observation. In chronic mild pulmonary tuberculosis an initial dose of $\frac{1}{20000}$ of a milligramme of T.R. may be given, gradually increasing to $\frac{1}{1000}$ In more active disease the initial dose should be $\frac{1}{100000}$. Good results are recorded from the Continent from much larger doses, *e.g.* even 1,000 milligrammes of T.O.A. If the dose is excessive quiescent disease becomes active, active disease more active.

Hort hopes shortly to have ready a method of measuring the reaction between antigen and antibody, and of thus producing a method of blood examination which will help the clinician to form a sound judgment as to the wisdom of employing tuberculin in any given case.

Krause,[2] working on the question of immunity to tuberculosis, draws several conclusions from his work on guinea-pigs. Among the main results of his investigations, he shows that guinea-pigs may be specifically sensitised by tuberculo-protein introduced by any parenteral route. These experiments on guinea-pigs further suggest that it may be quite possible to sensitise a non-tuberculous human subject with a von Pirquet, or intradermic or subcutaneous test, and that as a result of this the individual would later give a reaction to larger doses of tuberculin. Acute anaphylaxis was developed by toxic injections given post-orbitally or intravenously. Probably any of the products of the tubercle bacillus will sensitise guinea-pigs. The shortest incubation period noted has been six days, the longest two hundred and eighty-six, the size of the sensitising dose bearing no relation to the period of incubation. Once sensitiveness to the tuberculo-protein is acquired by a non-tuberculous animal, the repeated absorption of more protein tends only to increase the condition. Tolerance to tuberculo-protein cannot be attained in the non-tuberculous animal by gradually raising the dosage or diminishing the intervals of time between doses.

Very little work appears to have been done to establish the relations between hyper-sensitiveness to the products of a given micro-organism and susceptibility to infection from it. Experiments have, therefore, been carried out to ascertain whether guinea-pigs sensitised to tuberculo-protein would be more or less susceptible to experimental tuberculous infection than normal animals. The animals which were sensitised all became diseased, evidence which strengthens the opinion that sensitisation to a bacterio-protein does not carry with it an immunity to infection; further, hyper-sensitiveness and increased resistance, two different things, can co-exist in the same animal.

Other investigations on pulmonary tuberculosis carried out by Caulfield [3] and Beatty seem to show that certain classifications of the disease may be tentatively drawn by gauging the tuberculin sensitiveness, by using the reaction of complement fixation, and by certain opsonic observations. Possibly different tubercular subjects may overcome their infection along different biological paths.

From work done by Caulfield [4] on serum reactions in which the hæmolytic system consisted of sheep's corpuscles, anti-sheep rabbit serum and guinea-pig complement, it appeared that a positive test might be obtained upon a specific and non-specific basis. Tuberculous sera can be divided into two classes by their interaction with antigens derived from tubercle bacilli, and used in so-called anti-complementary strengths.

1. *Inhibition.*—Sera with this effect allow hæmolysis on the addition of the hæmolytic system because their sera-antigen resultants are repellant to complement (all clinical normals with positive tuberculin reactions and many suspects showed this in a more or less degree).

2. *Tuberculous sensitisers.*—Sera with these specific bodies cause the fixation of the complement, shown by complete absence of hæmolysis.

These biological classifications carry with them to a certain extent comparable clinical tendencies, and certain prognostic values attributable to the type of tuberculin reaction may be further narrowed and safeguarded.

The production of immunity by the inoculation of living tubercle bacilli has been attained in the guinea-pig by G. B. Webb and W. W. Williams. [5] An animal was inoculated weekly for nine months with a tubercle culture from which one hundred and fifty bacilli had been found to infect a guinea-pig. The inoculations were made subcutaneously, and began with two live bacilli, increasing to 5, 16, 20, 28, 40, 55, 75, 100, 150, 200 and so on, until at the end of seven months it had received 42,235 living bacilli and remained perfectly healthy. The animal was now tested with tuberculin according to the method of Römer, and gave a negative reaction. Inoculations were continued till the end of the ninth month, when 141,835 living bacilli had been injected. The animal was then killed owing to an epidemic of guinea-pig cholera. There were no tubercular lesions at the inoculation points, and no sign of tubercular infection elsewhere. Pieces of both lung apices, liver, spleen, kidney and one small inguinal gland were ground up in salt solution, strained through gauze and injected intraperitoneally into a guinea-pig. Seven months later this animal was killed, when no evidence of tuberculosis could anywhere be discovered.

A guinea-pig has therefore received, without any production of tuberculosis, about one thousand times a lethal dose of living virulent bacilli. Inoculations along similar lines are being carried out on monkeys.

**Multiple arthritis after the injection of tuberculin.** Hæmoptysis, broncho-pneumonia, pleural effusion, miliary dissemination of tuberculosis, angina pectoris, hemorrhagic nephritis and tetany may all result from the injection of large doses of tuberculin. Diem records a case[6] in which an injection of 0.5 mgm. of tuberculin T.R. was made in the right forearm. The following day the temperature rose to 102.2, the right forearm becoming œdematous and red. In the right wrist joint and in all the joints of the fingers much effusion appeared, the overlying skin becoming livid. The right axillary glands became enlarged and tender. The temperature became normal on the seventh day, and the swelling of the joints had disappeared on the tenth. The tuberculin was sterile and used without complications in other cases. The syringe had been kept in alcohol and the skin cleaned with alcohol. There was no sign of suppuration. It is interesting to note that all the affected joints were distal to the site of the injection.

J. R. Charles.

REFERENCES.

[1] E. C. Hort, *Quart. J. Med.*, 1911, v., 377.

[2] A. K. Krause, *J. Med. Research*, 1911, xxiv. 361.

[3] A. H. Caulfield and J. C. Beatty, *Ibid.*, 103.

[4] A. H. Caulfield, *Ibid.*, 101.

[5] G. B. Webb and W. W. Williams, *Ibid.*, 1.

[6] Diem, *München. Med. Wchnschr.*, 1911, lviii. 254; abstract in *Med. Rev.*, 1911, xiv. 377.

